# Vision-Based Human-Machine Interface for an Assistive Robotic Exoskeleton Glove

**DOI:** 10.21203/rs.3.rs-3300722/v1

**Published:** 2023-08-31

**Authors:** Yunfei Guo, Wenda Xu, Pinhas Ben-Tzvi

**Affiliations:** 1 Electrical and Computer Engineering Department, Virginia Tech, Blacksburg, VA, USA.; 2 Mechanical Engineering Department, Virginia Tech, Blacksburg, VA, USA.

**Keywords:** Human Machine Interface, Exoskeleton Glove Force Planning, Material Classification

## Abstract

This paper presents a vision-based Human-Machine Interface (HMI) for an assistive exoskeleton glove, designed to incorporate force planning capabilities. While Electroencephalogram (EEG) and Electromyography (EMG)-based HMIs allow direct grasp force planning via user signals, voice and vision-based HMIs face limitations. In particular, two primary force planning methods encounter issues in these HMIs. First, traditional force optimization struggles with unfamiliar objects due to lack of object information. Second, the slip-grasp method faces a high failure rate due to inadequate initial grasp force. To address these challenges, this paper introduces a vision-based HMI to estimate the initial grasp forces of the target object. The initial grasp force estimation is performed based on the size and surface material of the target object. The experimental results demonstrate a grasp success rate of 87. 5%, marking significant improvements over the slip-grasp method (71.9%).

## 1 Introduction

Exoskeleton gloves are used to restore the grasping ability to perform Activities of Daily Living (ADLs) for patients with brachial plexus Injuries (BPI) (Xu et al., 2020; Jian et al., 2018; Ge et al., 2020) or for post-stroke rehabilitation (Rahman and Al-Jumaily, 2012; Stilli et al., 2018; Sun et al., 2021; Iqbal and Baizid, 2015; Bauer et al., 2021). BPI is usually caused by motorcycle or snowmobile accidents that damage the neural system of the hand, resulting in lost mobility and sensation (Midha, 1997). Stroke, caused by disruption of blood flow to the brain, can damage the area of the brain that controls muscle movement, resulting in reduced mobility and sensation in the hand (Hunter and Crome, 2002). In both cases mentioned above, an exoskeleton glove is a promising solution to improve the quality of life for patients with hand disabilities.

In recent decades, numerous wearable robotic rehabilitation exoskeleton gloves have been developed to assist patients with hand disabilities (Xu et al., 2020; Jian et al., 2018; Ge et al., 2020; Rahman and Al-Jumaily, 2012; Stilli et al., 2018; Sun et al., 2021; Iqbal and Baizid, 2015; Bauer et al., 2021; Ma and Ben-tzvi, 2015; Ma and BenTzvi, 2015; Lee and Bae, 2015; Popov et al., 2017; Refour et al., 2019). Unlike robotic hands and grippers, which require a full automated grasping system, exoskeleton gloves require a semi-human guided control system. Patients who wear the exoskeleton glove will manually aim at the object during grasping. Also, the exoskeleton glove can only provide a limited number of degrees of freedom in terms of mobility, thereby limiting the types of grasps it can exhibit. Thus, human-machine interfaces (HMIs) for robotic exoskeleton gloves only need to determine the grasp type and force.

Various HMIs have been developed to control exoskeletons, including Electroencephalogram (EEG), Electromyography (EMG), vision, and voice-based, with each HMI having its advantages and disadvantages. An EMG-based HMI is the most commonly used method. It can be used to provide real-time motion and force planning directly from the wearer through EMG sensors placed on the forearm (Bronks and Brown, 1987; Artemiadis and Kyriakopoulos, 2008). Most researchers only used EMG sensors to detect gestures due to their good wearability (Chen et al., 2021; Cheon et al., 2020; Li et al., 2021; Yun et al., 2017; Lalitharatne et al., 2013; Huang et al., 2021). However, patients with paralysis of the hand have significantly weaker muscle EMG signals than normal people (Zhou et al., 2021). Therefore, EMG-based approaches are not suitable for patients with extremely weak or no hand function. The researchers designed multiple other HMIs to control the exoskeleton gloves. EEG-based HMI can provide a force planning feature (Paek et al., 2015) similar to the EMG approach, but suffers from wearability issues of the EEG sensor (Araujo et al., 2021; Li et al., 2019). Vision-based HMI requires minimal user action, but is low in precision and lacks initial grasp force planning ability (Kim et al., 2019; Pham et al., 2015; Ko et al., 2023; Calandra et al., 2018; Yamaguchi and Atkeson, 2017; Takamuku and Gomi, 2019). Voice-based HMI is known for its outstanding high accuracy, but lacks force planning ability (Guo et al., 2020; Wang et al., 2019; Kim et al., 2020). Force planning is critical for exoskeleton HMIs and can only be provided by the user through EMG-based HMIs. The lack of force planning ability will result in a slow and unstable grasp. Providing force planning on non-EMG-based HMIs has become one of the most challenging problems in exoskeleton glove control.

In this research, we focus on solving the aforementioned force planning issue by adding a vision-based HMI to a voice-controlled exoskeleton glove. Computer vision techniques are used to estimate the size, weight, and surface material of the target object. The estimated weight and size information is used to estimate the initial grasp force.

The main contributions of this study are summarized as follows. Initially, transfer learning was applied to state-of-the-art house interior surface materials detection techniques, adapting them to effectively identify materials on common objects in constrained contexts. Subsequently, a novel computer vision based HMI system was created, specifically tailored for assistive robotic exoskeletons. This inventive system tackles challenges in force planning by precisely estimating the dimensions, weight, and surface material of the target object. Lastly, grasp experiments were employed to showcase the effectiveness of the vision-based HMI in approximating the initial grasp force. The outcomes revealed a notably elevated success rate in grasping, surpassing that of the traditional slip-grasp method.

### 1.1 Exoskeleton Glove Hardware

**Fig. 1:**
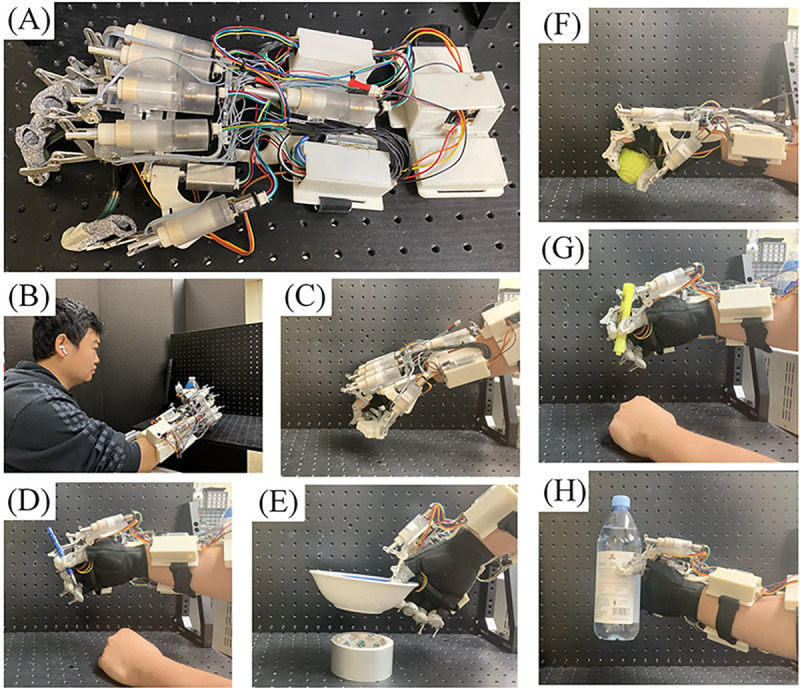
The assistive exoskeleton glove used in this research. This assistive exoskeleton glove is designed for patients with BPI. (A) Overview of the exoskeleton glove. (B) The user grasps a water bottle using voice-based HMI. (C) The user grasps a paper box with a tip grasp. (D) The user grasps a plastic pen with a tripod grasp. (E) The user grasps a ceramic bowl with a lateral grasp. (F) The user grasps a plastic ball with a sphere grasp. (G) The user grasps a plastic marker pen with a tripod grasp. (H) The user grasps a plastic bottle with a cylinder grasp.

This research employs an assistive exoskeleton glove tailored for patients with BPI (Xu et al., 2020, 2023). As individuals with BPI lack control over their muscles, this exoskeleton glove serves as a replacement for hand function. Key features of the exoskeleton glove include the utilization of Series Elastic Actuators (SEAs) alongside data-driven control and calibration for precise force measurement and control (Guo et al., 2021). The exoskeleton glove incorporates 7 SEAs to manage finger extension and contraction, thumb joint rotation, and wrist bending motion, enabling it to perform five rudimentary grasp types: cylinder grasp, sphere grasp, tip grasp, tripod grasp, and lateral grasp (as shown in Fig. 1). Each grasp type has been specifically designed to handle certain types of objects, as illustrated in Fig. 3. For instance, the cylinder grasp is well-suited for grasping water bottles and cups, while the tip grasp is ideal for handling spoons and forks.

The exoskeleton’s operation can be outlined in three steps. First, the user interacts with the exoskeleton through a voice-based Human-Machine Interface (HMI) to instruct it on the desired grasp type (Guo et al., 2020, 2022). Second, the user, having a functional arm, selects an appropriate grasp position based on the object’s location and places the exoskeleton accordingly. Third, force planning is carried out using a slip-grasp force planning method to adjust the grasp force (Guo et al., 2022; Xu et al., 2022). However, this method encounters challenges due to sensor limitations, as discussed in the related work section (Sec. 2.1). To address this issue, a vision-based HMI is proposed, which estimates the object’s size and weight, thus aiding in the force planning of the exoskeleton.

## 2 Related Work

### 2.1 Limitations of Force Planning Methods used for Exoskeleton Gloves

Previous research proposed several methods to solve the force planning problem in non-EMG-based HMIs. However, force planning strategies suffer from two problems, as described below.

First, exoskeleton gloves need to grasp objects with unknown shapes, surface material, and weight. Nevertheless, all force planning algorithms require the setting of equations with precise grasp position, friction coefficient, and weight to calculate the optimal contact forces. Vanteddu et al. developed two methods to satisfy two of the conditions required for a stable grasp. These include deformation prevention of soft objects and maintaining force and moment equilibrium of the objects being grasped. Like exoskeleton gloves, some robotic hands and grippers also face the same problem. Cheng and Orin used the compact-dual linear programming method to find the force distribution for a robotic grasping system called DIGITs. Youshen Xia et al. proposed using recurrent neural networks for grasp force optimization for multi-fingered robotic hands. Xiong and Xiong used an algorithm based on an artificial neural network to determine the joint torques that must be applied to a multifingered robotic hand required for a successful grasp. However, during normal usage of assistive exoskeleton gloves, the grasping position, object weight, surface material, and object size are almost impossible to determine accurately, thus making the above algorithms difficult to use.

Second, exoskeleton gloves need to predict the grasp force before lifting the object. Previous researchers designed a slip-grasp method to find the appropriate force through trial and error. Lee et al. proposed a slip detection method using a customized pressure sensor to measure slippage at the fingertips of the SAFER exoskeleton glove. A hybrid slip detection method for an exoskeleton glove was proposed by Xu et al.. This method utilizes both Serial Elastic Actuators (SEA) and pressure sensors to enhance its accuracy. The force controller adds force to the fingertips if the object slips. However, the reinforcement process typically results in a tedious grasping process in which the user must continue to find the optimal grasp force through failures, which is not practical for exoskeleton glove users. Moreover, slip detection on a robotic exoskeleton glove differs from a robotic hand or gripper due to space and size limitations. Previous researchers have designed multiple slip detection sensors for robotic hands and grippers and have achieved good results in the slip-grasp force planning method (Romeo and Zollo, 2020; James and Lepora, 2020). However, there is not enough space for larger and more accurate slip detection sensors to be fitted at the fingertips in an exoskeleton glove application. The limitation of sensors makes the slip-grasp method suffer from accuracy issues.

### 2.2 Vision-Based Force Planning on Exoskeleton Gloves

Researchers have previously performed extensive research on vision-based force planning using robotic grippers. Pham et al. used a computer vision system to estimate the pose of the hand and object to assist in force planning. However, their research assumed that the weight of the object is known. Similarly, most vision-based grasping methods focused on position estimation to assist force planning (Yu et al., 2013; Liu et al., 2019; Zhang et al., 2021). Ko et al. and Takamuku and Gomi used the RGB camera to predict the grasp force based on the motion of the object. Their methods are used mainly to improve the synchronicity between the grasp and load forces. However, their methods do not provide an initial prediction of the grasp force. Calandra et al. and Yamaguchi and Atkeson designed vision-based reinforcement learning methods to predict the optimal initial grasp force. However, their method shares performance issues similar to the slip detection methods. Initial estimation of the grasping force remains an ongoing research challenge.

Humans can grasp and lift an object without knowing its exact weight, surface material, and size. Studies have shown that even with restricted haptic feedback, humans can still perform a stable grasp based on visual input (Stone and Gonzalez, 2015). Humans can use vision to estimate the grasp force. If the object’s actual size, weight, and surface friction coefficient match the estimation, the predicted force will be close to the optimal grasp force. Haptic feedback is used to detect slippage when the estimated force is inaccurate. Humans can adjust the grasp force according to the haptic feedback.

Humans can perform accurate force planning even with restricted haptic feedback. Researchers working on the development of exoskeletons have attempted to capture these biological signals from force planning using EMG or EEG methods to assist force planning (Bronks and Brown, 1987; Artemiadis and Kyriakopoulos, 2008). However, these methods require conversion of the user’s intention to biological signals to create control outputs, which suffer from low signal-to-noise ratios, significant processing time, and long reaction times. This research is inspired by the human force planning method. Instead of capturing the EEG or EMG signal, this paper proposes a computer vision-based HMI that mimics a human grasping procedure to directly estimate the size, weight, and surface material of an object and can calculate the initial grasp force based on static force analysis.

### 2.3 Material Recognition in the Wild and MINC-2500 Dataset

Surface material detection using computer vision is the key to solving the aforementioned force planning issues. Weight can be estimated based on the surface material, and the surface friction coefficient can be directly acquired. The state-of-the-art material detection datatset is MINC-2500. Bell et al. built the MINC dataset with images of human-labeled material in the real world and proposed a deep learning-based material segmentation method. This method uses a convolution neural network to generate a probability map and the conditional random field (CRF) algorithm to calculate a label for each pixel. The advantage of this method is that it does not require a pixel-wise label, which is ideal for applications with limited segmented data. MINC-2500 is a subset of the MINC dataset, which contains 57,500 image patches for 23 different types of materials. However, the MINC-2500 dataset mainly contains long-shot (LS) or extra-long-shot (ELS) interior design images, which are taken from a distance and contain many different objects in context. This research focuses on detecting the surface material of objects in images that are taken in a close-up (CU) or medium-close-up (MCU) view. Transfer learning was performed to transfer the learned weight from MINC-2500 to the collected dataset to improve the accuracy of material classification. The setup of the neural network and the experimental results are discussed in Sec. 5 and Sec. 8.4.

## 3 HMI System Overview

The vision-based force planning method is designed to grasp an object without the need for detailed measurements in advance. The goal is to find the initial grasp force by estimating the size, shape, weight, and surface material of the object to be grasped.

This vision-based initial grasp force estimation method uses voice input from a microphone to initiate grasping and releasing (input voice command: “grasp” and “release”). Such a voice command system is proposed by Guo et al. (2022). After receiving a grasp command, the camera embedded in the glasses will start to take pictures and perform the following three steps on the image to calculate the initial grasp force.

**Fig. 2:**
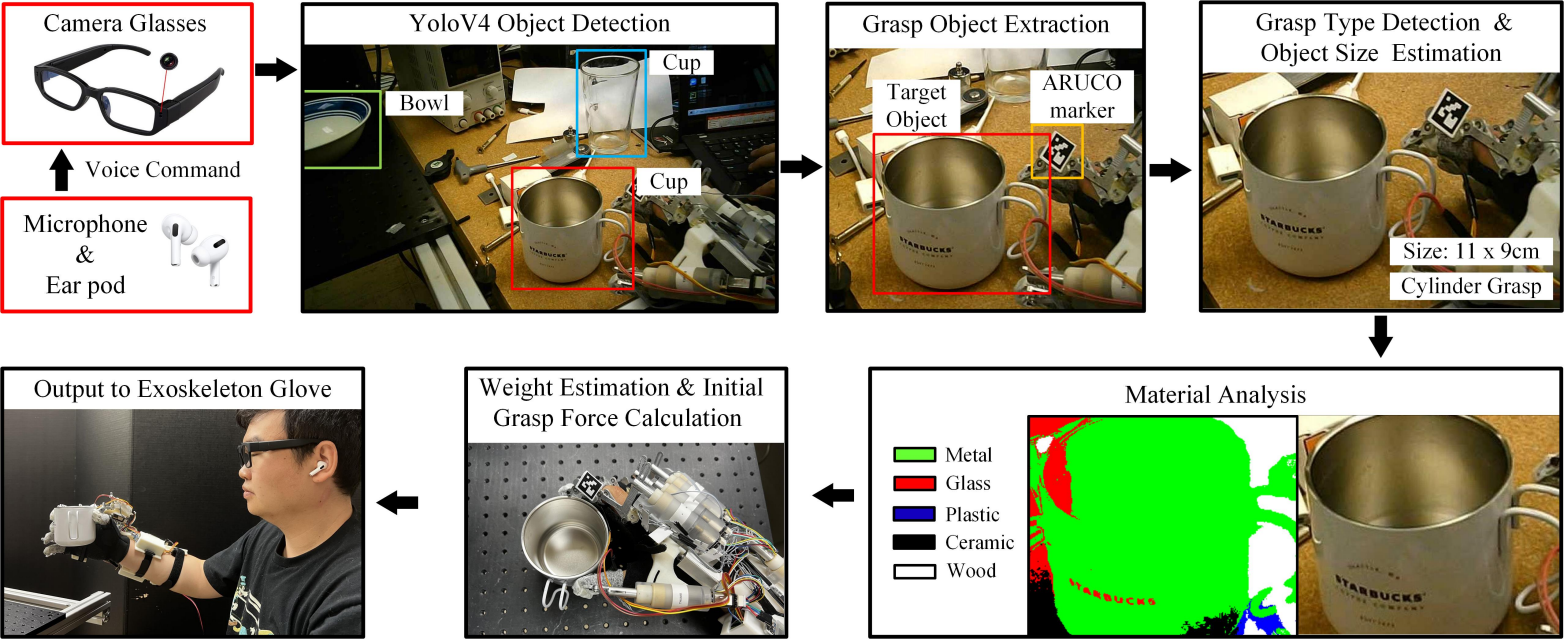
Overview of vision-based initial grasp force prediction procedure.

1. The input images are sent to an object detector trained on the Common Objects in Context (COCO) dataset. This step will help the vision-based force planning method to understand the environment by detecting all objects in the view and extracting the target object using an ARUCO marker on the exoskeleton glove (ARUCO marker is shown in Fig. 2). In this step, the target object category and size are acquired and the grasp type is determined according to the target object’s category.
2. The surface material of the target object is acquired by performing a material classification or material segmentation on the image patch of the target object. Given the object’s size and surface material, the object’s weight can be estimated.
3. The initial grasp force is calculated based on the spatial location of the exoskeleton, the surface material of the target object, and the weight.

**Fig. 3:**
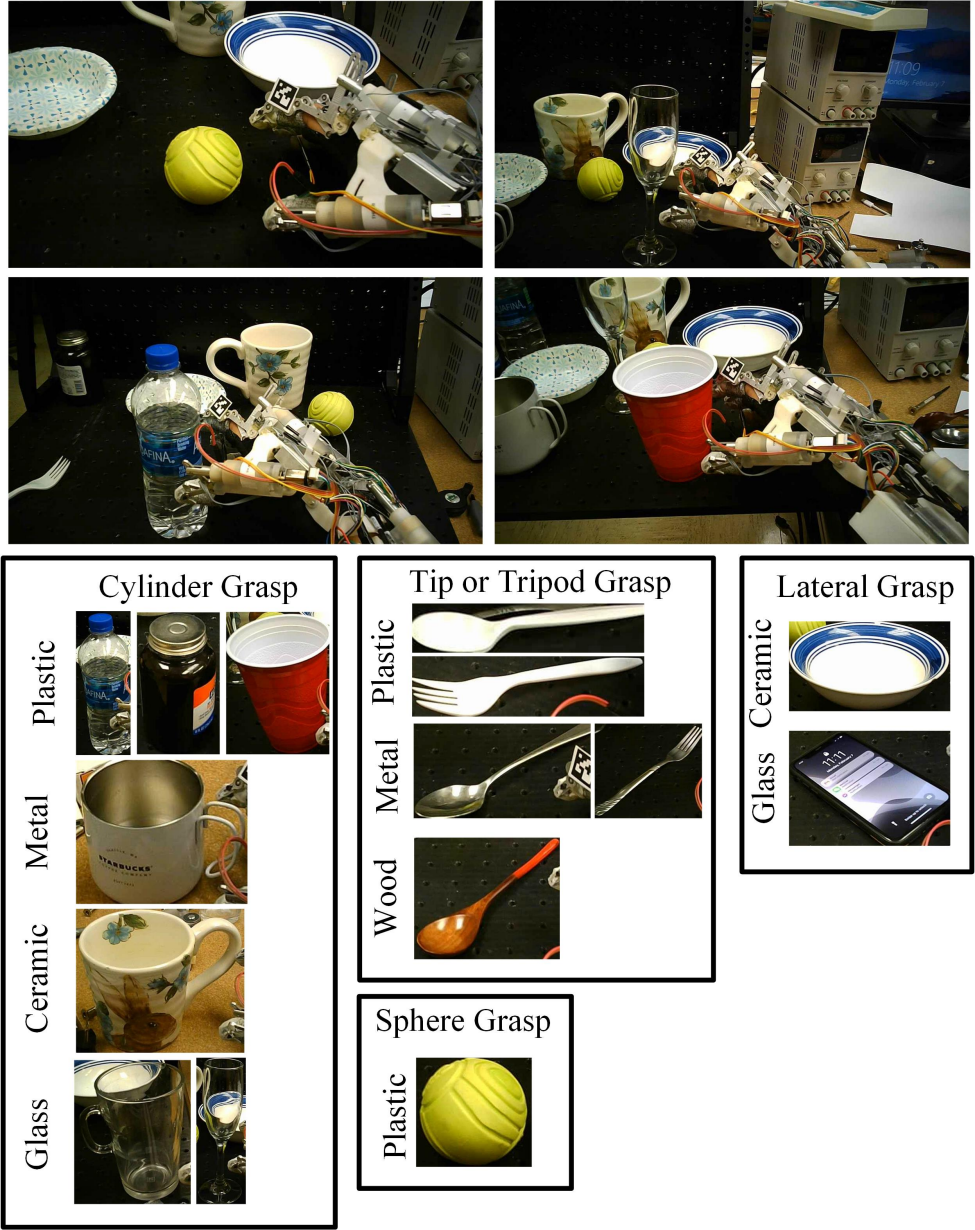
Sample images for the exoskeleton grasping environment, object category, and object material.

The initial grasp force is then sent to the exoskeleton. The SEAs are FSRs on the exoskeleton glove will detect slip while applying the predicted initial grasp force, and the slip-grasp method will adjust the grasp force as needed. The structure of the vision-based force planning method is shown in Fig. 2. Sample images for the exoskeleton grasping environment, object category, and object material are shown in Fig. 3.

### 3.1 HMI System Characteristic

The proposed HMI has the following characteristics:

1. The vision HMI is designed specifically for human-guided assistive robotic exoskeleton gloves. In this application, the location where the object is located in reference to the location of the glove is controlled by the user, and the vision HMI can generate initial grasp force to help the exoskeleton grasp target objects.
2. The initial grasp force generated by the HMI is not the optimal grasp force. For example, a non-transparent plastic cup full of water and an empty plastic cup shows no difference in the proposed vision-based estimation system. The estimation system can set a range for the initial grasp force that is not too far from the optimal grasp force to help the system grasp the object.
3. The vision HMI can generalize to detect objects only in the MS COCO dataset because the object detector is trained using MS COCO. Material detection can generalize to detect the surface material of different objects but may be limited to contexts. This system cannot detect the new material category without training.

## 4 Object Detection

There are two common approaches for detecting and locating an object in an image: object detection (Gao et al., 2020; Chen et al., 2019; Girshick, 2015; Tan et al., 2020) or instance object segmentation (Liang et al., 2018; Siddique et al., 2021). Object detection requires image annotation using a bounding box during training. The detection result for object detection is a bounding box that contains background information. Thus, object detection is faster during training and inference. Instance object segmentation requires pixel-wise image annotation for training, and the detection result consists of pixels of the object without backgrounds. Object segmentation can better understand the object’s shape, but is slower during training and inference than object detection. In this research, object detection was used over object segmentation for two reasons.

1. Object detection is faster than object segmentation during inference proccess. Two-stage object segmentation will first detect the object in a bounding box and then extract the object pixels from the background. Single-stage object segmentation uses a decoder network to find the object and an encoder to propagate the object’s pixels. Both methods mentioned above need additional calculations during inference, thus being slower than object detection using bounding boxes. The need for speed in this application necessitated the use of object detection instead of object segmentation.
2. Object detection techniques have better data availability. Object detection does not necessitate pixel-level labeling, and this study may address the difficulty of grasping items that are not included in publicly accessible datasets. To detect uncommon objects in a small-scale project, transfer learning or fine-tuning on a public dataset is usually employed. Therefore, object detection techniques are utilized in this research as they require less annotation and will have better data availability.

The state-of-the-art object detection methods are based on Single Shot Detector (SSD) (Chen et al., 2019), Faster R-CNN (Girshick, 2015), EfficientDet (Tan et al., 2020), and YOLOV4 (Gao et al., 2020). Researchers have previously tested these methods on the COCO dataset (Lin et al., 2014). The inference speed and Mean Average Precision (mAP) at 50% Intersection over Union (IOU) of seven different object detection methods are compared on the collected validation dataset in order to select the most suitable object detection method. Sample images of the collected validation dataset are shown in Fig. 3. The experimental results are shown in Fig. 8. According to the experiments, YOLOV4 was selected as the object detection method used in this research; it better balanced speed and mAP than other methods.

### 4.1 Size Estimation for Target Object

The data output from object detection will be an object category vector c, an object bounding box vector B, and an object center vector S. The *n*^*th*^ object detected in an image belongs to category ${\;}^{n}c$.


(1) $$c=\left[{\;}^{1}c,{\;}^{2}c,\ldots ,{\;}^{n}c\right]$$

For the *n*^*th*^ object detected in an image, the object’s bounding box ${\;}^{n}b$ is the combination of the upper left corner ${\;}^{n}p_{ul}=\left({\;}^{n}x_{ul},{\;}^{n}y_{ul}\right)$ and the lower right corner ${\;}^{n}p_{lr}=\left({\;}^{n}x_{lr},{\;}^{n}y_{lr}\right)$.


(2) $$B=\;\left[{\;}^{1}b,{\;}^{2}b,\ldots ,{\;}^{n}b\right]\;\;=\left[\left({\;}^{1}x_{ul},{\;}^{1}y_{ul},{\;}^{1}x_{lr},{\;}^{1}y_{lr}\right),\ldots ,\left({\;}^{n}x_{ul},{\;}^{n}y_{ul},{\;}^{n}x_{lr},{\;}^{n}y_{lr}\right)\right]$$

For the *n*^*th*^ object detected in an image, the center of the pixel of the detected object is located at ${\;}^{n}s$ calculated from the bounding box ${\;}^{n}b$.


(3) $$S=\;\left[{\;}^{1}s,{\;}^{2}s,\ldots ,{\;}^{n}s\right]\;\;=\left[\left({\;}^{1}x_{s},{\;}^{1}y_{s}\right),\ldots ,\left({\;}^{n}x_{s},{\;}^{n}y_{s}\right)\right]$$

The target object is selected on the basis of the distance to the ARUCO marker located on the exoskeleton glove. The output of the ARUCO Application Programming Interface (API) contains the center coordinate of the marker: $s_{m}=\left(x_{m},y_{m}\right)$.

The exoskeleton glove used in this research is right-handed with the ARUCO marker placed on the index finger linkage (see Fig. 3). The object to be grasped is likely to be on the lower right of the ARUCO marker. A weighted distance function was customized to find the distance between the ARUCO marker center coordinate $s_{m}$ and the detected *n*^*th*^ object center ${\;}^{n}s$:

(4) $${\;}^{n}d=w_{0}\left(x_{m}-{\;}^{n}x_{s}\right)+w_{1}\left({\;}^{n}y_{s}-y_{m}\right)\;\;+\sqrt{{\left(x_{m}-{\;}^{n}x_{s}\right)}^{2}+{\left(y_{m}-{\;}^{n}y_{s}\right)}^{2}}$$

where, ${\;}^{n}d$ is the *n*^*th*^ object distance between the object center and the ARUCO marker center. $w_{0}$ is the weight that serves as the penalty for the object located on the right of the marker, and $w_{1}$ is the weight that serves as the penalty for the object located above the marker. $\left({\;}^{n}x_{s},{\;}^{n}y_{s}\right)$ is the coordinate of the center of the object from the vector of the center of the object ${\;}^{n}s$. The grasped object’s index i can be found by minimizing the customized distance function ${\;}^{n}d$:

(5) $${\;}^{i}d=min\left({\;}^{1}d,{\;}^{2}d,\ldots ,{\;}^{n}d\right)$$

The category of the target object is ${\;}^{i}c$, the bounding box is ${\;}^{i}b$, and the center coordinate is ${\;}^{i}s$.

### 4.2 Finding the Target Object Size using ARUCO Marker

Theoretically, it is not possible to obtain the exact size of an object without using a stereoscopic camera. However, it was assumed that the ARUCO marker and the target object have the same distance from the camera. Thus, the size of the target object can be estimated on the basis of the size of the ARUCO marker.

**Fig. 4:**
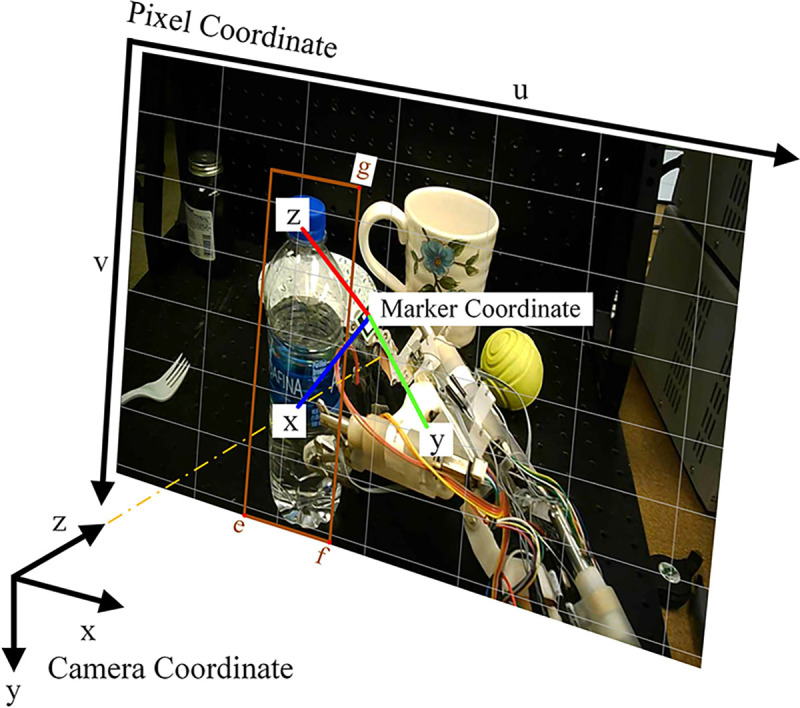
Illustration of the camera, marker, and pixel coordinates.

The marker width and height are 2 centimeters. The coordinates are explained in Fig. 4. The coordinates of the detected object’s bounding box ${\;}^{i}b$ can be transferred from pixel coordinates to camera coordinates, and then to marker coordinates. The Euclidean distance between the points e and f in the marker coordinates is the length of the object (w) in centimeters (the points are shown in Fig. 4). The Euclidean distance between points f and g in the marker coordinates is the height of the object (h) in centimeters.

The following method can be used to convert points from pixel coordinates to marker coordinates. The ARUCO API outputs the rotation vector (r) in the axis-angle representation, and the center coordinate (t) of the marker in the camera coordinates. To transfer a point $p_{p}=(u,v)$ from the pixel coordinates to the camera coordinates $p_{c}=\left(x_{c},y_{c},z_{c}\right)$, the following equations are used:

(6) $$x_{c}=\frac{u\;-\;s_{x}}{f_{x}}d_{z}$$

(7) $$y_{c}=\frac{v\;-\;s_{y}}{f_{y}}d_{z}$$

where, $d_{z}$ is the distance from the marker to the camera in the camera coordinates. $s_{x}$ and $s_{y}$ are the coordinates of the principle point in the camera coordinates (640 and 360 in this application). $f_{x}$ and $f_{y}$ are focal lengths of x and y axes in pixels (1184 and 1249 in this application).

To transfer a point $p_{c}=\left(x_{c},y_{c},z_{c}\right)$ from the camera coordinate to the marker coordinate $p_{m}=\left(x_{m},y_{m},z_{m}\right)$, the following equations are used:

(8) R=Rodrigues(r)

(9) $$p_{m}=R^{T}\left(p_{c}-t\right)$$

where, Rodrigues formula was used to build a transformation matrix R from the axis-angle representation rotation vector r.t is the marker coordinate center represented in the camera coordinates.

## 5 Material Classification

There are two common approaches to detect the surface material of an object, including image classification based on center pixels and semantic segmentation on the entire image (Bell et al., 2015; Zhang et al., 2017; Zhao et al., 2017). The most widely used material classification datasets are the Flicker Material Dataset (FMD), MINC, and open surface datasets. There are only limited pixel-wise annotated images provided, and most of these annotated images are furniture from the interior of a house, which is very different from this application. Due to the limited availability of annotated data, a pixel-wise supervised classification method such as UNet (Siddique et al., 2021; Zhao et al., 2017) cannot be used. For this application, the center pixel classification method was used to classify the material of a given object image, and the conditional random field (CRF) (Krähenbühl and Koltun, 2011) method was used for segmentation. Material segmentation is used to visualize the classification result.

Since this application focuses on grasping daily used objects as shown in Fig. 3, the number of classes in MINC-2500 was reduced from 23 to 5, which include ceramic, metal, glass, plastic, and wood.

### 5.1 Material Classification Challenges

Initially, the deep learning material classification method was trained and tested on MINC-2500 and achieved good accuracy. The original MINC dataset material patch classification was trained on VGG-16, AlexNet, and InceptionV1 in 2014. The VGG-16 architecture was used as a performance baseline to test the new networks, which achieved high classification accuracy in the ImageNet challenge: InceptionResNetV2 and ResNet152V2. Moreover, networks that achieve similar classification accuracy were tested, but have faster inference speeds: InceptionV3, ResNet50V2, and MobileNetV2. In addition to different network architectures, the NetVLAD pooling method was tested, which is a clustering-based pooling method commonly used in speaker verification, face detection, and place recognition (Arandjelovic et al., 2016).

The weight of the model is transferred from ImageNet, and the training is terminated if the validation loss does not decrease for ten consecutive epochs. The training result was tested on a small data set similar to the use case of this application, which contains images from the FMD dataset and images collected online. Some sample images from the data can be visualized in Fig. 7. The dataset contains 169 images for each of the five categories.

The training results and model performance comparison are shown in Tab. 1. According to training results, ResNet50V2, MobileNetV2, and Inception V3 are the top 3 networks that achieve a good time and performance balance in the MINC-2500 validation set. However, the MINC-2500 does not have a perfect generalization to material classification. The context in the MINC dataset is very different from that of this application, which prevents the network from finding a correct label during testing on the collected dataset. NetVALD clustering pooling layer also does not improve accuracy. To solve the generalization issue, transfer learning was performed to retrain the model in the collected dataset. Transfers from ImageNet and MINC-2500 weight were experimented. The results are shown in Tab. 2. The results show that the transfer from MINC-2500 using ResNet50V2 has the best accuracy when testing on the collected dataset.

### 5.2 Proposed Approach: Transfer Leaning using ResNet50V2

**Fig. 5:**
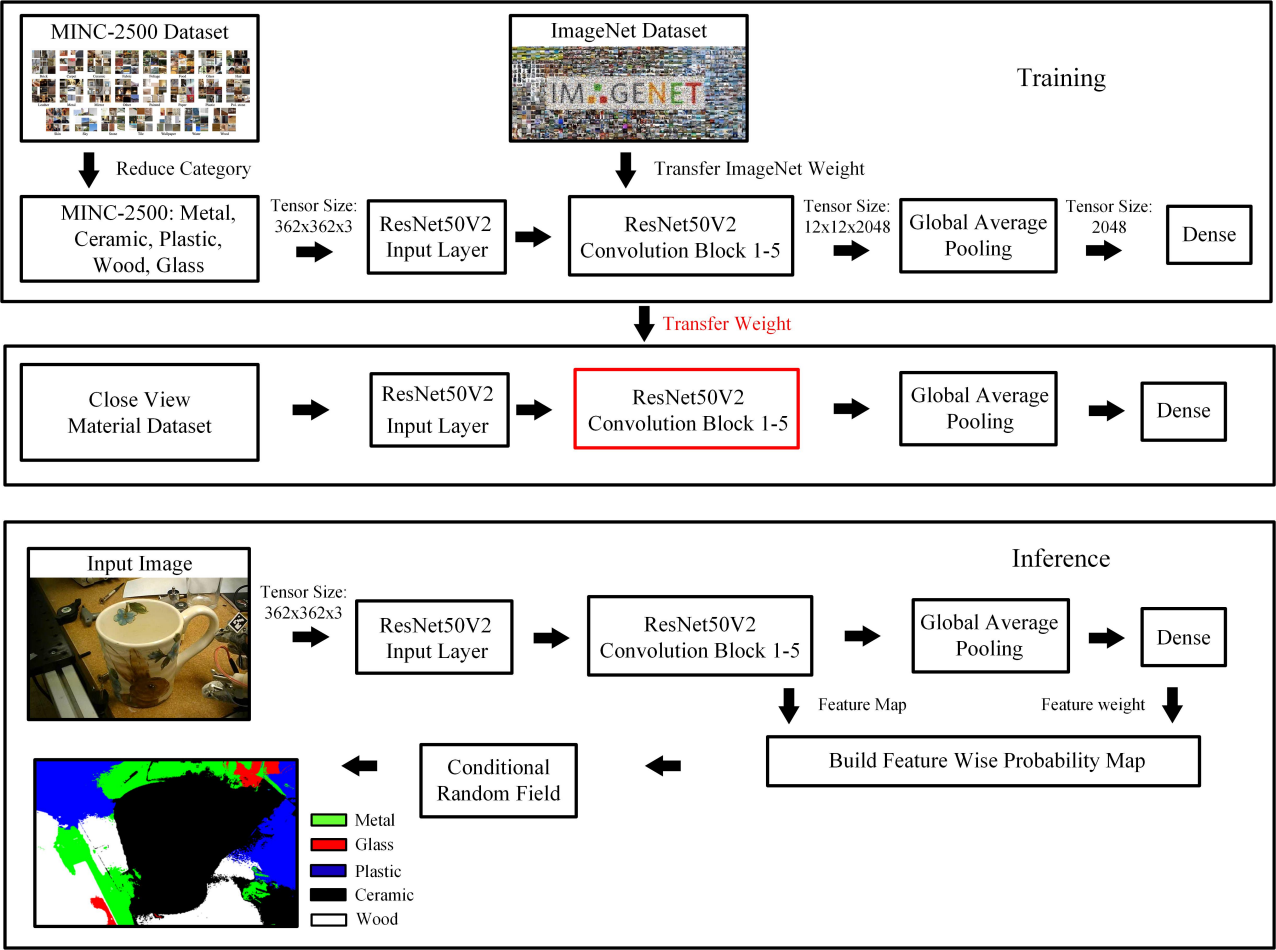
Training and inference procedure for vision-based material classification and segmentation.

Based on the experimental results from the previous section, ResNet50V2 was used to transfer the weight from ImageNet to the MINC-2500 dataset. The number of material classes in MINC-2500 is reduced to metal, ceramic, plastic, glass, and wood. The input layer is modified to match the MINC-2500 size, the convolution blocks from ResNet50V2 have not been modified, and the weight is trained using the initial value from ImageNet. The output of the convolution layer consists of 2048 feature maps $M_{\left[12\times 12\times 2048\right]}$. The pooling layer uses global average pooling to group the feature maps $M_{\left[12\times 12\times 2048\right]}$ to $M_{\left[1\times 1\times 2048\right]}$ and classified into five classes multiplied by weight $W_{\left[5\times 2048\right]}$. Due to the low generalization accuracy of the MINC-2500 data set, the MINC-2500 weight was transferred to the collected dataset using the same architecture. The training and inference procedure is shown in Fig. 5.

When inferring on a sample image, the ResNet50V2 network was modified to output a class probability map ${\;}_{c}P_{\left[1\times 5\right]}$ and a feature-mapsized class probability map ${\;}_{f}P_{\left[12\times 12\times 5\right]}$ using GradCAM (Selvaraju et al., 2017). The Grad-CAM is generated using the following equation:

(10) $${\;}_{f}P=\sum_{n=1}^{2048}{\;}^{n}W^{n}M$$

Where, ${\;}^{n}M$ is the *n*^*th*^ feature map and ${\;}^{n}W$ is the weight of the *n*^*th*^ feature map. The probability map ${\;}_{f}P_{\left[12\times 12\times 5\right]}$ will be resized to pixel level probability map ${\;}_{p}P_{\left[362\times 362\times 5\right]}$ using cubic spline interpolation. The probability map ${\;}_{p}P_{\left[362\times 362\times 5\right]}$ and colored image $I_{\left[362\times 362\times 3\right]}$ are input into a Conditional Random Field (CRF) algorithm to perform pixel level unsupervised segmentation by minimizing the following energy function (Krähenbühl and Koltun, 2011):

(11) $${}^{c}E\left(x\right)=\sum_{i}U\left(i\right)+\sum_{\left(i,j\right)}Par\left(i,j\right)$$

where, ${\;}^{c}E(x)$ is the energy function for class c.x is the set of all pixels in image I.i and j are pixel indexes in set x.i and j control a nested loop to pair each pixel with all other pixels without repetition. U(i) is the unary energy that is the negative log probability of a pixel belonging to class c.Par⁡(i,j) is the pairwise energy that measures the pixels’ spacial and color similarity. The unary and pairwise energy is defined in the following equations:

(12) $$U(i)=-log\left({\;}_{p}^{i}P_{c}\right)$$

(13) $$Par(i,j)=exp(-\frac{{\left|{\;}^{i}p\;-{\;}^{j}p\right|}^{2}}{2s_{p}^{2}}-\frac{{\left|{\;}^{i}I\;-{\;}^{j}I\right|}^{2}}{2s_{c}^{2}})$$

where, ${\;}_{p}^{i}P_{c}$ is the pixel level probability of *i*^*th*^ pixel in the image belonging to class $c.\;{\;}^{i}p$ and ${\;}^{j}p$ are the position of *i*^*th*^ and *j*^*th*^ pixels. ${\;}^{i}I$ and ${\;}^{j}I$ are the RGB values of *i*^*th*^ and *j*^*th*^ pixels. Long-range connections were used in the energy calculation. Thus, the pairwise energy contains only the appearance kernel. $s_{p}$ and $s_{c}$ are the position similarity and color similarity parameters, respectively. Parameter values $s_{p}$ and $s_{c}$ were chosen to be 60 and 10 respectively based on Krähenbühl and Koltun. The results of the CRF algorithms will be an updated pixel level probability map ${{\;}_{crf}P}_{\left[362\times 362\times 5\right]}$.

The classification results can be found by finding the maximum value of the ${\;}_{c}P$ class probability map. The results can be directly used to estimate the grasp force. The segmentation results can be used to perform pixel-wised classification when the target object contains different materials. The sample segmentation results and classification accuracy are available in Sec. 8.4.

## 6 Weight Estimation

The estimated size and material of the target object can be obtained based on the methods described in the previous sections. However, the information is insufficient to estimate the weight, and some assumptions need to be made in order to calculate the volume of the target object.

The target object in this application can be classified into four different categories: fork/spoon, bottle/cup/wine glass, sports ball, and apple/cell phone. The weight of an apple and a cell phone is not affected much based on size; thus, the average weight of an apple and a cell phone can be used as the weight of the target object. Sports balls are usually very light, so it was assumed that a sports ball weighs 20 grams if it has a diameter less than 5cm, weighs 100 grams if it has a diameter between 5–10cm, and weighs 250 grams if the diameter is larger than 10cm.

The shape of a spoon or fork can be simplified to a plate with a thickness of 0.1 cm. Thus, the weight of a spoon or fork can be estimated using the following:

(14) $$v_{sf}=0.1wh$$

(15) $$s_{sf}=v_{sf}\rho$$

where, w and h are the estimated width and height of the target object, respectively. ρ is the density of the material of the target object. $v_{sf}$ is the volume of the object. $s_{sf}$ is the weight of the target spoon or fork.

The shape of a bottle, cup, and wine glass can be simplified to a hollow truncated cone. It is assumed that the truncated cone has $\frac{2}{3}$ of the volume of a cylinder of the same height. The thickness can be assumed to be 0.2cm. Thus, the weight of a bottle when filled with water can be estimated using the following.

(16) $$v_{b}=\frac{2}{3}\left(v_{o}-v_{i}\right)\;\;=\frac{2}{3}(\pi {\left(\frac{w}{2}\right)}^{2}h-\pi {\left(\frac{w}{2}-0.2\right)}^{2}(h-0.4))\;$$

(17) $$s_{b}=v_{b}\rho +v_{i}\rho_{w}$$

where, $v_{b}$ is the volume of the material to form the bottle. $v_{o}$ is the outer volume, $v_{i}$ is the inner volume. $s_{b}$ is the weight of the bottle. ρ is the density of the material of the bottle. $\rho_{w}$ is the density of water.

The weight of a cup can be estimated similar to that of a bottle. The only difference is that a cup might have a handle and will make the volume calculation inaccurate. The size of the handle was assumed to be 30% of the weight of the cup w. Thus, the weight of a cup when full of water can be estimated using the following.

(18) $$\begin{array}{l} \text{if}\;h\geq w\;:\\ \;v_{c}\;=\frac{2}{3}(v_{o}-v_{i})\\ \;=\frac{2}{3}(\pi {\left(\frac{w}{2}\right)}^{2}h-\pi {\left(\frac{w}{2}-0.2\right)}^{2}(h-0.2)) \end{array}$$

(19) $$\begin{array}{l} if\;w\geq h\;:\\ \;v_{c}\;=\frac{2}{3}(v_{o}-v_{i})\\ \;=\frac{2}{3}(\pi {\left(\frac{0.7w}{2}\right)}^{2}h-\pi {\left(\frac{0.7w}{2}-0.2\right)}^{2}(h-0.2)) \end{array}$$

(20) $$s_{c}=v_{c}\rho +v_{i}\rho_{w}$$

where, $v_{c}$ is the volume of material to form the cup. $v_{o}$ is the outer volume, and $v_{i}$ is the inner volume. $s_{C}$ is the weight of the bottle. ρ is the density of the material of the cup. $\rho_{w}$ is the density of water. Wine glass is a special cup with a long leg, so it was assumed that the capacity of the glass is 50% of a normal cup. Thus, the weight of a wine glass when full of water can be estimated using the expression:

(21) $$s_{wg}=v_{c}\rho +0.5v_{i}\rho_{w}$$

## 7 Initial Grasp Force Calculation

**Fig. 6:**
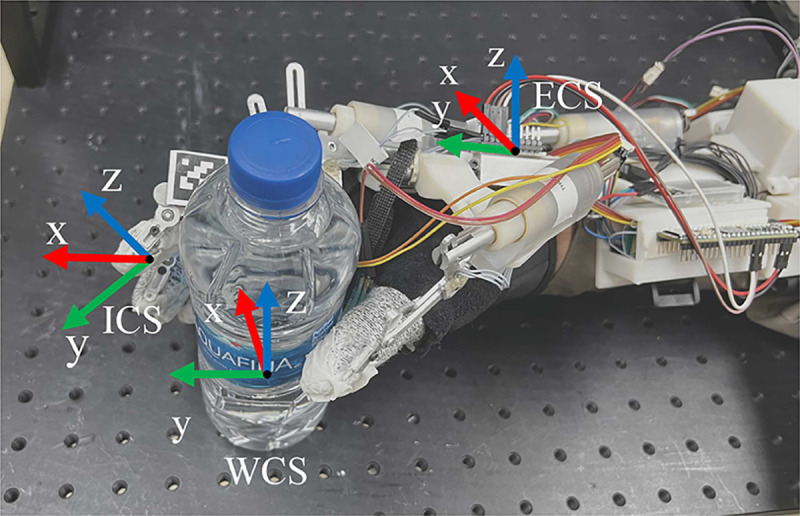
The coordinate systems for initial force estimation. WCS: world coordinate system. ECS: exoskeleton glove coordinate system. ICS: *i*-th fingertip coordinate system.

The initial grasp force is calculated based on the predicted weight and the shape of the standard object. Fig. 6 illustrates the coordinate systems for grasping force initialization. The origin of the world coordinates is placed at the center of the object. The exoskeleton glove coordinates are located at the center of the Inertia Measurement Unit (IMU). The IMU is calibrated to align with the world coordinates at the beginning. Assuming that there is no torque applied on the object and the contact forces are normal to the last link of each of the exoskeleton fingers, for an arbitrary object, the force equilibrium equation can be expressed as:

(22) $$\sum_{i}\mu^{w}R_{e}{\;}^{e}R_{i}{\;}^{e}F_{i}+Mg=0$$

where, i∈{thumb,index,middle,ring,little}, ${\;}^{w}R_{e}$ is the rotation matrix from the exoskeleton glove coordinates to the world coordinates, which is calculated based on readings from the IMU. ${\;}^{e}R_{i}$ is the rotation matrix from the fingertip i to the exoskeleton glove coordinates, which is calculated based on the forward kinematics of the glove (Xu et al., 2020). ${\;}^{e}F_{i}$ is the vector of the contact force applied on fingertip i, which is measured based on a calibrated Linear Series Elastic Actuator (LSEA) (Guo et al., 2021). M is the mass of the object, and g is the vector of gravitational acceleration.

For the cylinder grasp and the tip grasp, the direction of the friction force on each fingertip is always opposite to gravity. Therefore, the above equation can be simplified to $\sum_{i}\;\mu F_{i}=Mg$.

## 8 Experimental Results

The experiment section encompassed three primary components. Initially, the datasets utilized for object detection validation and material classification were introduced. Subsequently, the performance of object detection, size estimation, and material classification within these datasets was assessed. Lastly, a vision-based HMI was integrated as an extension of the slip-grasp force planning method for the exoskeleton glove. The experiments were structured to contrast the combined approach of vision and the slip-grasp method against the exclusive use of the slip-grasp force planning method.

### 8.1 Datasets

Two small datasets were built to verify this application (object detection dataset); one for vision-based grasp force planning method validation and one for transfer learning material classification (material classification dataset).

The dataset for vision-based grasp force planning method validation has 30 images taken from 1080P SVWSUN Video Glass worn by an exoskeleton glove user. Each grasp object is labeled using a bounding box. Sample images are shown in Fig. 3.

**Fig. 7:**
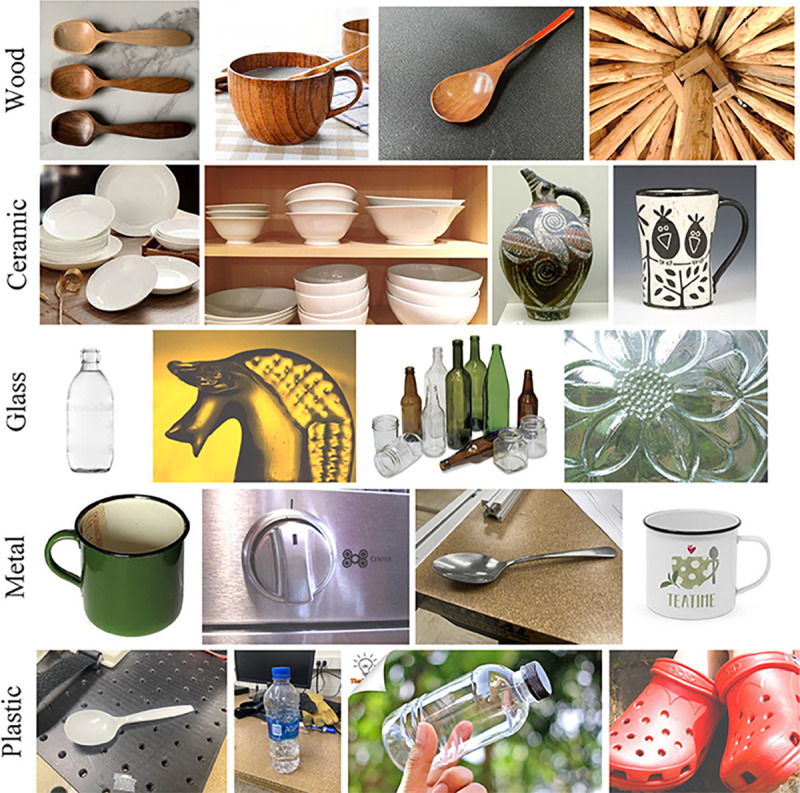
Sample images used in the material classification training.

The dataset for transfer learning consists of five labels: ceramic, plastic, metal, wood, and glass. Each class has a training set of 119 images, a testing set of 30 images, and a validation set of 20 images. Each image is labeled on the basis of the object’s center material. This dataset contains images from an online image search, the FMD dataset, and images taken for the grasp objects used in this research. Sample images are shown in Fig. 7. Images in this dataset have more details and fewer contexts than images in MINC-2500.

### 8.2 Object Detection and ARUCO Marker Detection

**Fig. 8:**
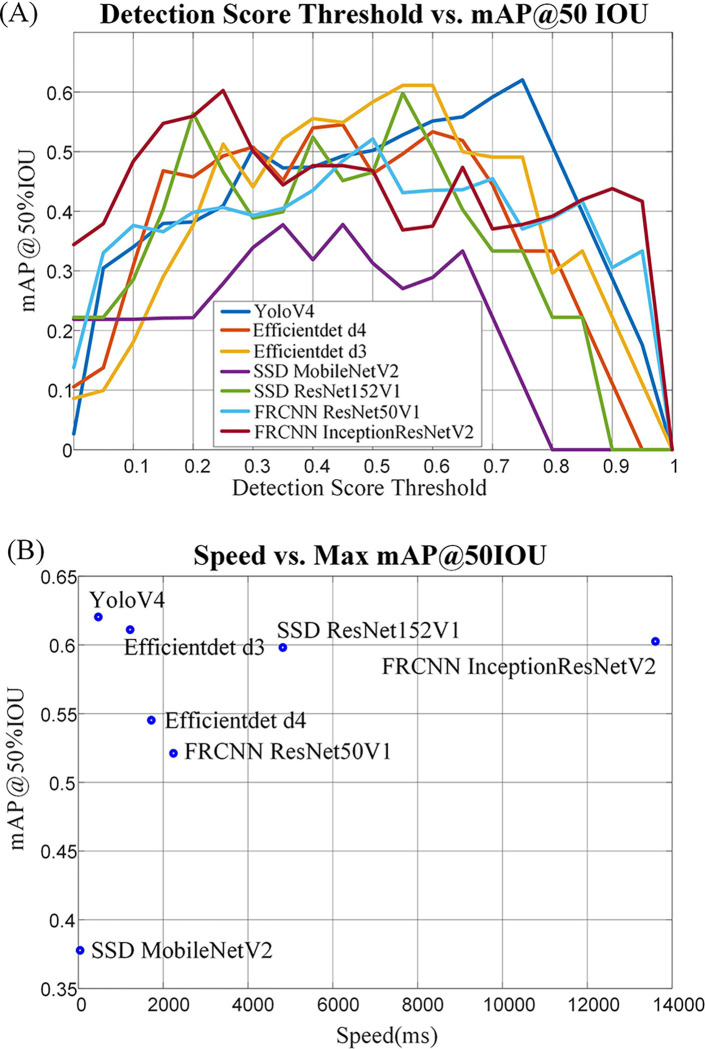
Object detection results. (A) mean Average Precision (mAP) at 50% Intersection over Union (IoU) of 7 different state-of-the-art neural networks. (B) mAP vs. average inference time of each neural network.

The labeled object detection validation dataset was used to test the performance of different networks trained on the COCO dataset. A mean Average Precision (mAP) at 50% Intersection over Union (IOU) was used to quantify object detection performance. The speed was measured based on the average inference time of 10 images using the E5–1260 CPU. The results are shown in Fig. 8. Multiple networks were tested and YOLOV4 with a 0.75 threshold was selected based on mAP and speed.

The successful detection rate $R_{s}$ of object detection and ARUCO marker detection can be calculated using the following equation:

(23) $$R_{s}=\frac{TP\;-\;FP}{n}$$

where, TP is true positive, which means that the ARUCO API detection successfully detects the marker, and the object detection successfully identifies the center object. FP is false positive, which means that the marker detection recognized the wrong marker or the object detection detects the wrong center object. n is the total number of test images. The experiments’ successful detection rate was 90% in the collected object detection validation dataset.

### 8.3 Object Size Estimation

**Fig. 9:**
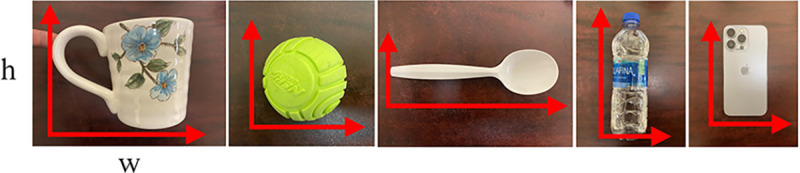
Examples of size measurements.

The experiment involved evaluating the object detection validation dataset by comparing the detected target object’s size with the ground truth sizes. For this purpose, images successfully detected by both the YoloV4 object detector and the ARUCO marker detector were utilized. This dataset comprised 27 images featuring 15 different objects observed from various angles. To obtain the predicted size for each object, the average of the estimated sizes from different angles was taken. The ground truth sizes were determined based on the width and height of the orthographic projection, as illustrated in Fig. 9.

The obtained results are presented in Tab. 3. To quantify the difference between the predicted and actual object sizes, the percentage difference between the products of width (w) and height (h) was calculated. This evaluation metric is termed the Mean Absolute Percentage Error (MAPE). The MAPE difference between the predicted and actual object sizes was found to be 26.9%. The main source of this error was identified as the estimation process, particularly when utilizing the bounding box to estimate the object’s dimensions. This error tends to occur when the object is placed at an angle during detection.

### 8.4 Object Material Detection

The training and testing results in the proposed material classification dataset are shown in Tab. 2. According to the accuracy and speed of classification, the material classification network used is ResNet50V2. The weight is transferred from the MINC-2500 dataset.

**Fig. 10:**
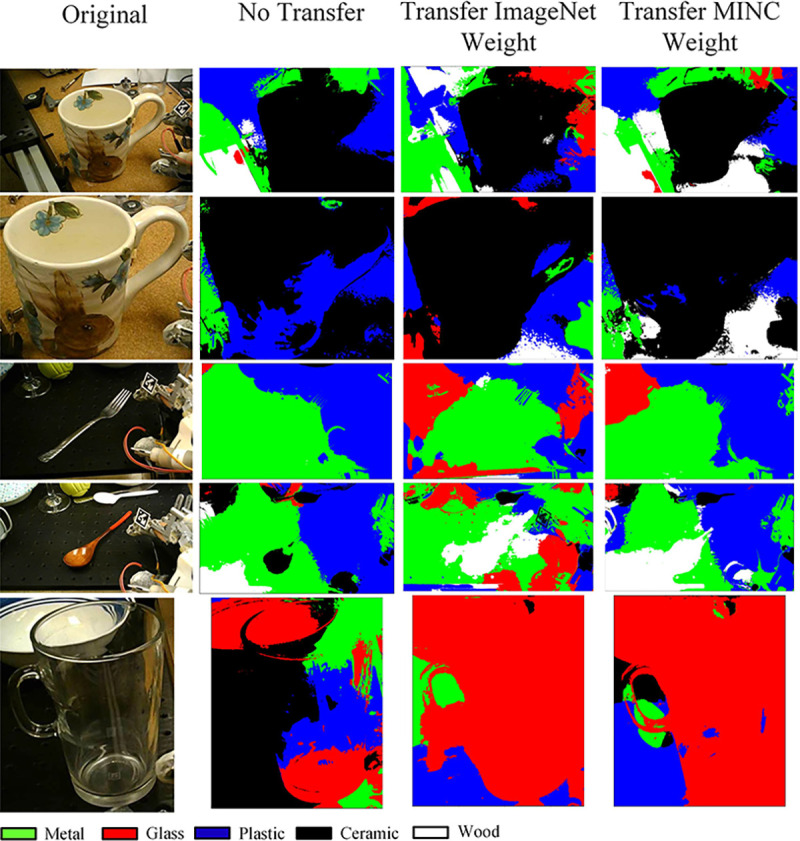
Sample material segmentation results.

Material classification validation was also performed on the object detection dataset. The material classification accuracy for all detected objects was 96%. In addition to material classification, material segmentation is performed using the CRF method to visualize the result of material classification. Sampe images of material segmentation are shown in Fig. 10.

### 8.5 Object Weight Estimation

The experiments on the object detection validation dataset involved comparing the weight of the target object with the weight of the corresponding ground truth. The dataset comprised 27 detected images used in the size estimation process, which relied on the estimated sizes obtained in the previous section. The materials used in the objects had different densities: plastic (0.92*g*/*cm*^3^), metal (7.85*g*/*cm*^3^), glass (2.7*g*/*cm*^3^), ceramic (6*g*/*cm*^3^), and wood (0.9*g*/*cm*^3^).

The results of these experiments are presented in Tab. 4. However, it is worth noting that the weight of the containers varied due to differences in the fluid level. For consistency, it was assumed that all containers were full. To assess the accuracy of the weight estimation, Mean Absolute Percentage Error (MAPE) was employed as the evaluation metric. The MAPE between the predicted and actual object weights was found to be 59.8%. The relatively large weight estimation error can be attributed to the following factors. First, weight estimation is heavily influenced by size estimation, which in turn can be affected by the angle at which the object appears in the camera. Second, the assumption of standard shapes for all objects, such as cylinders or boxes, may not hold true for most cases, where cups might have handles, and wine glasses may have long legs, leading to deviations from the standard shapes used in the estimation process. Furthermore, despite some instances of substantial percentage errors, the overall weight difference remains acceptable. For instance, the metal fork experienced a weight estimation error of 35g, representing a 159.1% overestimation compared to its actual size. The average weight difference across all objects is only 173g, which still provides meaningful information for initial grasp force planning.

### 8.6 Grasp Experiments

The experimental procedure involving human subjects in this study received approval from the Carilion Clinic Institutional Review Board (IRB-19-330). Due to the nature of the exoskeleton glove used in this research, which is a rigid linkage exoskeleton, the user cannot apply any force to the fingertips of the exoskeleton linkages when wearing it.

The grasp procedure is as follows: The user initiates the system using a personalized voice command system (Guo et al., 2020) to capture a 1280×760 pixel image. By employing the methods proposed in previous sections, the size and weight of the grasped object can be calculated. The 9-DOF MPU-9250 IMU detects the pitch, yaw, and roll of the exoskeleton glove using an AHRS filter. Using the weight of the object and the IMU data, the initial grasp force is computed, and the exoskeleton glove applies this force to each fingertip (Guo et al., 2021). The slip-grasp system is then utilized to stabilize the grasp.

During the experiment, each of the 15 objects present in the object detection dataset was subjected to 2–6 grasping attempts from various angles and water levels (for containers), resulting in a total of 64 grasp trials. Among these trials, 6 experienced failure of object detection, while 5 encountered errors in material detection. The grasp success rate is defined as the success in picking up the target object. The overall grasp success rate using vision-based HMI combined with the slip-grasp method was 87.5%.

### 8.7 Comparison Between Vision-based Force Estimation and Slip Grasp Force Planning

**Fig. 11:**
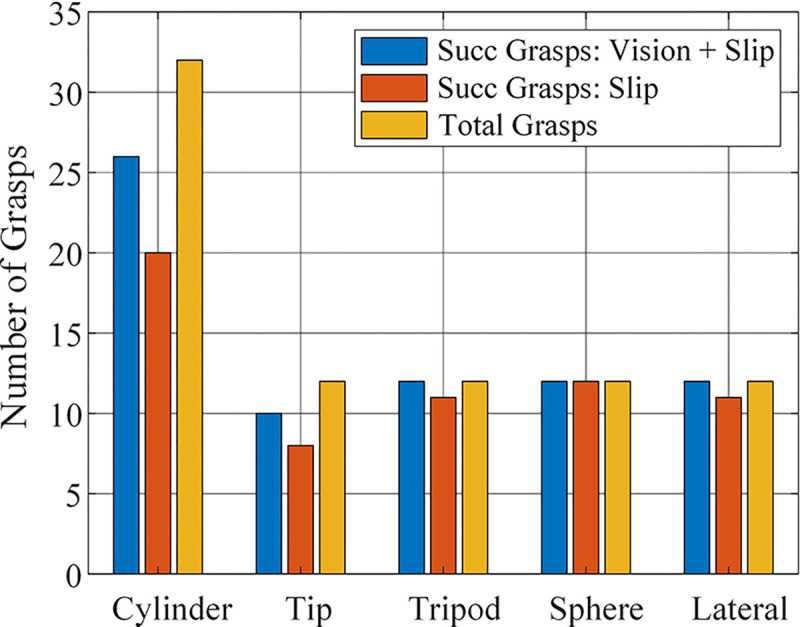
Experimental result of grasping daily used objects using vision-based initial grasp force prediction method and slip-grasp method. Blue: number of successful grasps performed using the vision-based initial force estimation with slip-grasp method. Red: number of successful grasps performed using only the slip-grasp method. Yellow: the total number of grasps for each individual method.

To demonstrate the effectiveness of the vision-based force estimation method. We performed 64 experiments using only the slip-grasp force planning method and achieved a grasp success rate of 71.9%, while the vision-based method achieved 87.5%. The success rate for each grasp category is shown in Fig. 11.

The comparison experiment reveals that utilizing a combination of vision-based force estimation with the slip-grasp system leads to a higher success rate compared to using only the slip-grasp system. To demonstrate the benefits of utilizing the vision-based initial force estimation technique, we carried out an additional set of 20 grasp trials involving four distinct items: a plastic bottle, a wine glass, a plastic spoon, and a metal spoon. These particular objects were chosen based on their notable performance in previous grasp experiments.

For the vision-based method, the initial grasp force was determined using the vision-based force estimation system, and the slip-grasp method was not utilized in this experiment. For the slip-grasp method, a predefined initial grasp force of 2N and 200Nmm is used. This method adjusted the grasp force based on slippage to achieve a stable grasp (details can be found in paper by Xu et al. (2022)).

**Fig. 12:**
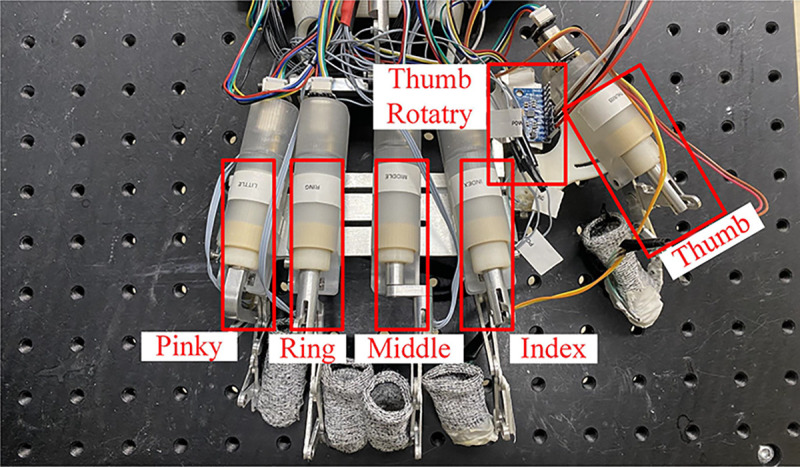
Series Elastic Actuators (SEA) are used to apply force on the exoskeleton glove in the grasp experiment.

The grasping process was facilitated by 6 Series Elastic Actuators (SEAs) as depicted in Fig. 12. The force and torque output of the index finger and thumb rotatory SEAs, which are the most critical actuators during grasping, were measured and reported in Tab. 5.

**Fig. 13:**
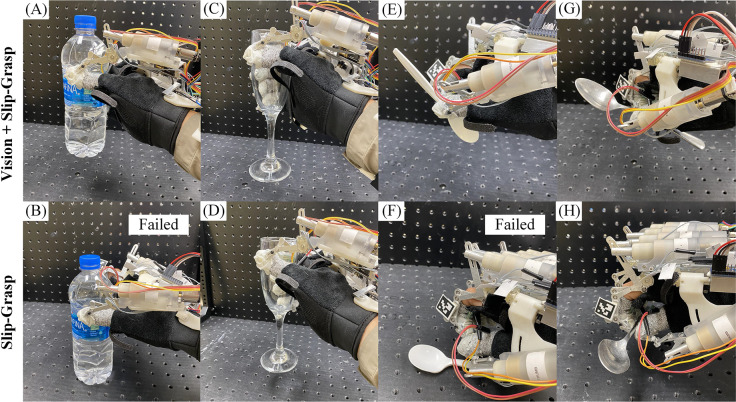
Demonstration of grasping daily used objects using vision-based initial grasp force prediction method and slip-grasp method. (A) Successfully grasp a 512g water bottle with vision system. (B) Failed to grasp a 512g water bottle using the slip-grasp method due to inadequate thumb torque. (C) and (D) Successfully grasp an 188g wine glass with both the vision system and the slip-grasp method. (E) Successfully grasp a 3g plastic spoon with vision system. (F) Failed to grasp a 3g plastic spoon using the slip-grasp method due to excessive force and torque. (G) and (H) Successfully grasp a 48g metal spoon with both the vision system and the slip-grasp method.

The results from the additional 20 grasp experiments are presented in Tab. 5 and Fig. 13, demonstrate that the vision-based force estimation system can produce adequate initial grasp forces for various objects. This offers three main advantages during grasping. First, the initial grasp force estimate helps prevent the application of insufficient thumb torque, which can result in slippage. For example, in Fig. 13 (B), the plastic water bottle could not be lifted by the slip-grasp method due to the insufficient predefined thumb torque. Second, the initial grasp force can prevent the application of excessive force and torque. For example, in Fig. 13 (F), the plastic spoon could not be lifted by the slip-grasp method due to excessive fingertip force and thumb torque. Third, even for objects that can be successfully lifted by the slip-grasp method, incorporating a vision-based force estimation system allows for a reduction in the applied force (as shown in Tab. 5), thereby optimizing the grasping process.

### 8.8 Vision-based HMI System Latency

The image processing is running on a desktop server with an E5–1260 CPU, and there is no GPU involved. The estimated size, weight, and surface friction coefficient are sent to the exoskeleton’s onboard microcontroller, which generates the initial grasp force using IMU data and operates the exoskeleton. The computation time for processing a single image is around 700 ms. The processing time meets this application’s requirements as only one image needs to go through the complete processing per grasp. The time consumption for processing one image is shown in Tab. 6.

## 9 Conclusion

This paper presented a novel vision-based Human-Machine Interface (HMI) aimed at estimating the initial grasp force required to manipulate a target object using an assistive exoskeleton glove designed for patients with Brachial Plexus Injuries.

The proposed approach employed object detection and material classification techniques to predict the initial grasp force, using information about the weight, size, and material of the object. In the validation dataset, the object size estimation produced a mean absolute percentage error (MAPE) of 26.9%, while the object weight estimation showed a MAPE of 59.8%. Although the MAPE of weight and size estimation was relatively high, vision-based initial grasp force estimation still managed to produce a meaningful result to assist grasping.

The vision-based HMI successfully distinguished between different materials and accurately predicted the initial grasp force for objects of varying weights. When integrated with the pure slip-grasp method, the combined approach attained an impressive 87.5% success rate, outperforming the standalone slip-grasp method (71.9%). These results highlighted the importance of estimating the initial grasp force to prevent slippage caused by inadequate or excessive application of force and torque.

In conclusion, the proposed vision-based HMI demonstrated the potential to enhance the grasping capabilities of the exoskeleton glove, contributing to improved functionality and usability for patients with Brachial Plexus Injuries. The findings of this experiment pave the way for future advancements in assistive technologies, facilitating more effective and reliable interactions between users and robotic systems.

## Figures and Tables

**Table 1:** Results of training on MINC-2500 and testing on the collected dataset

Network	MINC-2500 Acc	Collected dataset Acc	Speed (ms)*
VGG-16	71%	22%	279
**InceptionV3**	**83%**	21%	**215**
VGG-16-N*	68%	21%	292
InceptionV3-N*	77%	22%	225
**MobileNetV2**	75%	20%	**173**
**ResNet50V2**	78%	**23%**	228
ResNet152V2	84%	22%	487
InceptionResNetV2	81%	21%	472

a Speed*: the inference time is measured by inference of one image on a E5-1260 CPU.

b -N*: NetVALD layer with 32 clusters is added after the last convolution layer

**Table 2:** Performance comparison between transfer ImageNet and MINC-2500 weight to the collected dataset

Network	Transfer MINC-2500 Accuracy	Transfer ImageNet Accuracy
**ResNet50V2**	**79%**	76%
MobileNetV2	72%	71%
InceptionV3	75%	72%

**Table 3:** Size Estimation Experimental Results

Object	Actual* (cm)	Predicted* (cm)	Diff
Plastic Bottle A	6.5×11	8×13	45.4%
Plastic Bottle B	7×20	8×21	20%
Plastic Spoon	13×4	14×3.5	5.8%
Plastic Fork	14×4	14×3.5	12.5%
Plastic Cup	12×10	14×11	28.3%
Plastic Ball	7×7	6.8×6.8	5.6%
Metal Spoon	18×3.5	14.1×6.7	49.9%
Metal Fork	18×2.5	12×6	60%
Metal Cup	14×9	13.4×10.6	12.7%
Wood Spoon	16.5×4	11×7.8	30%
Glass Cup	12×14	12.9×15.1	15.9%
Wine Glass	20×5.5	19.4×7.3	28.7%
Ceramic Cup	19×11.5	18.8×15.2	30.8%
Ceramic Bowl	17.5×17.5	17×10	44.5%
Cell Phone	15×7.5	12×8.2	12.5%
MAPE	-	-	26.9%

a Actual*: The actual size is defined by the width times height in centimeters.

b Predicted*: The predicted size is defined by the width times height in centimeters.

**Table 4:** Weight estimation experimental results

Object	Actual(g)	Predicted(g)	Diff
Plastic Bottle A	12–512	698	36.3%*
Plastic Bottle B	207	432	108.7%
Plastic Spoon	3	5	66.7%
Plastic Fork	3	5	66.7%
Plastic Cup	11–502	881	75.5%*
Plastic Ball	69	100	45%
Metal Spoon	48	74	54.2%
Metal Fork	22	57	159.1%
Metal Cup	172–576	619	7.5%*
Wood Spoon	7	8	14.3%
Glass Cup	358–779	1459	89.5%*
Wine Glass	188–369	399	8.1%
Ceramic Cup	480–1059	1756	65.8%*
Ceramic Bowl	315	596	89.2%
Cell Phone	222	200	10%
MAPE	-	-	59.8%

*: Containers have various weight due to the content. During weight estimation, we assume all containers are full of water.

**Table 5:** Comparison between vision-based force estimation and slip grasp force planning

Object	Slip-Grasp (succ/total trials)	Vision (succ/total trials)	Slip (index force/thumb torque)	vision (index force/thumb torque)
Plastic Bottle	3/6	5/6	3.67N / 367Nmm	2.73N / 459Nmm
Wine Glass	6/6	6/6	2.67N / 267Nmm	1.59N / 267Nmm
Plastic Spoon	2/4	4/4	2N / 200Nmm	0.75N / 31.6Nmm
Metal Spoon	3/4	4/4	2N / 200Nmm	1.2N / 50.8Nmm

**Table 6:** Inference speed of one 1280×760 pixel image using the vision-based HMI

Section	Speed*(ms)
ARUCO marker Detection	7
Object Detection	470
Material Classification	228
Size and Weight Estimation	3
Total	708

Speed*: the inference time is measured by averaging the inference time of ten images on a E5-1260 CPU.
